# Metastatic renal Ewing’s sarcoma in adult woman: Case report and review of the literature

**DOI:** 10.1515/med-2021-0207

**Published:** 2021-03-09

**Authors:** Giovanni Cochetti, Alessio Paladini, Jacopo Adolfo Rossi de Vermandois, Sonia Fatigoni, Magda Zanelli, Stefano Ascani, Ettore Mearini

**Affiliations:** Department of Surgical and Biomedical Sciences, Urology Clinic, University of Perugia, Perugia, Italy; Department of Surgical and Biomedical Sciences, Medical Oncology, University of Perugia, Perugia, Italy; Department of Oncology and Advanced Technologies, Pathology Unit, Arcispedale Santa Maria Nuova di Reggio Emilia, AUSL-IRCCS di Reggio Emilia, Reggio Emilia, Italy; Department of Experimental Medicine, Institute of Pathologic Anatomy, “Santa Maria” Hospital, Terni, Italy

**Keywords:** extra-skeletal Ewing sarcoma, adult Ewing sarcoma, PNET, renal tumor, surgical margins

## Abstract

Primary renal extra-skeletal Ewing sarcoma is a rare neoplasm, often metastatic at diagnosis, and with a poor outcome. A multimodal approach is often the treatment of choice in this aggressive neoplasm. We present a case of primary renal extra-skeletal sarcoma in a 45-year-old woman who underwent tumor resection without clear margins. After no response to the first cycle of chemotherapy, we documented an early onset of local recurrence. The patient refused any other treatment and died four months after surgery.

## 1 Introduction

The Ewing Sarcoma (ES) family represents a group of high-grade malignancy tumors including ES of bone, Extra-skeletal Ewing sarcoma (EES), peripheral neuroectodermal tumor (PNET), and Askin tumor (a thoracopulmonary PNET). The ES family is a group of poorly differentiated tumors made up of small round blue cells and it recognizes the rearrangement of the ESWR1 gene as a pathognomonic sign [1]. The ES breakpoint region (ESWR1) maps on 22q12 and is one of the most involved genes in sarcoma, firstly identified in the ES family, but also present in other neoplasms.

ES is the second most common pediatric bone tumor, with a peak of incidence between the first and second decade and 80% of cases arise from the skeleton [2]. In adults, the most frequent primary presentation is an EES [3], which accounts for about 5% of all soft tissue sarcomas [1]. EES has no pathognomonic symptoms or signs and the clinical features depend mainly on the primary site. Potentially, EES could affect everywhere as single or multiple lesions [4]. The most common primary sites include the paravertebral spaces, lower extremities, head, neck, and pelvis. Sites rarely involved are the retroperitoneum, omentum, orbit, skin, and chest wall. The imaging features of EES are non-pathognomonic as well as clinical manifestations. It often presents as a well-defined, heterogeneous mass with areas of hemorrhage or necrosis in absence of calcification and nodal metastases [3]. EES can rarely arise in the kidney [4], resembling a renal cancer often with involvement of renal vein, inferior vena cava, or adjacent organs [3]. Most common metastatic sites in EES are lung, bone, and brain [3]. Therapeutic strategy of EES is debated. About 40–50% of the patients already present metastasis at diagnosis, so the role of the different therapies is yet to be defined. Multimodal treatment (surgery, radiotherapy, chemotherapy) determines an improvement in prognosis for single primary lesion [2], beside a poor prognosis for metastatic disease.

Herein, we report a case of an adult woman affected by an EES treated with surgery and chemotherapy. Secondary aim was to perform a review of the literature about EES and its therapeutic management.

## 2 Material and methods – case report

**Figure 1:**
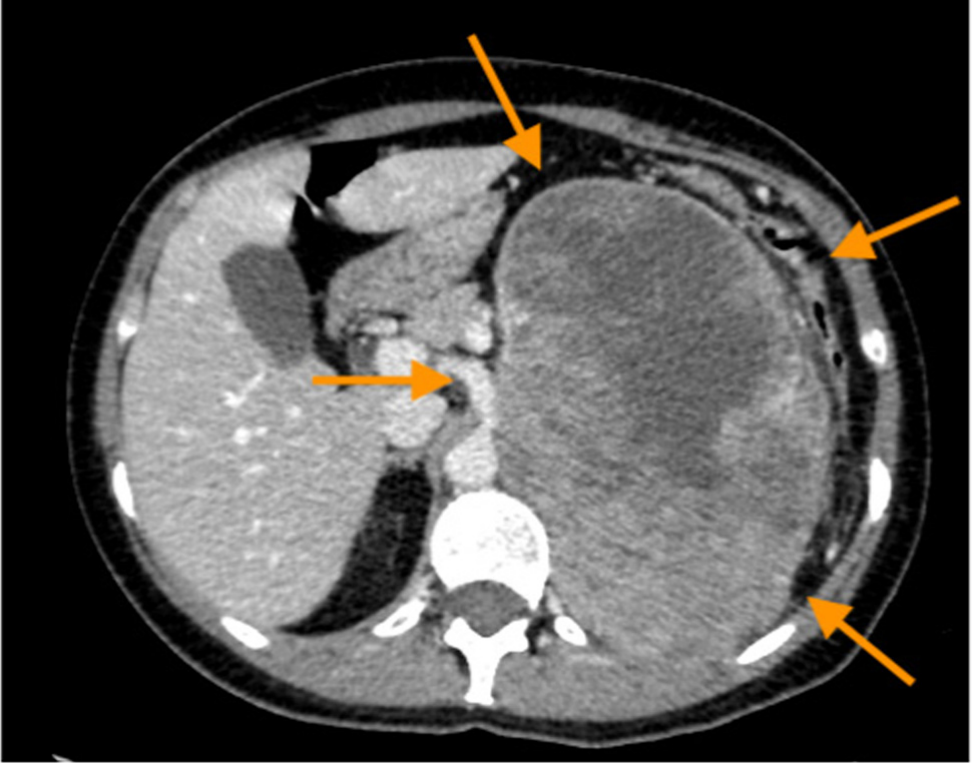
CT-scan, the imaging revealed a heterogeneous retroperitoneal mass of 17 cm in diameter containing necrotic and hemorrhagic areas, at the level of the superior left renal pole infiltrating partially the diaphragm (arrows).

**Figure 2:**
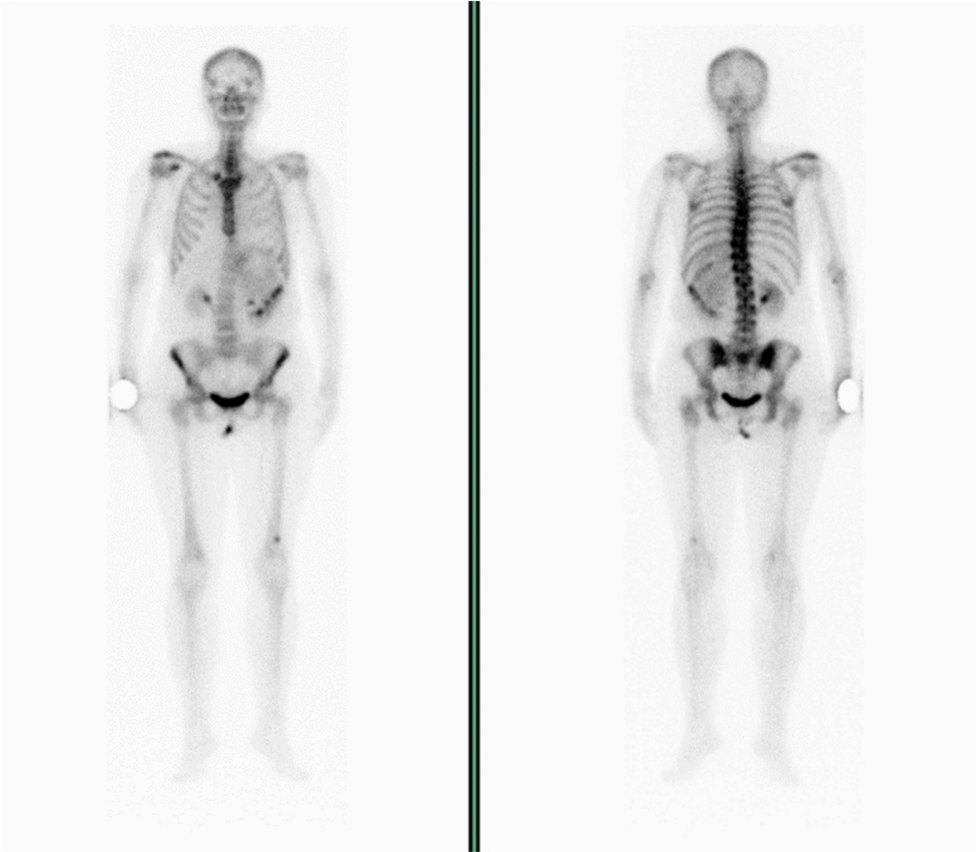
Total body bone scan performed for staging revealed no bone metastasis.

A 45-year-old female with persistent chest pain underwent chest-abdomen CT scan for suspicion of pulmonary thromboembolism. The imaging revealed a heterogeneous retroperitoneal mass, sized as 17 cm in diameter, extending from the superior left renal pole and infiltrating posterior abdominal wall and partially the diaphragm (Figure 1). The tumor was associated with neoplastic thrombosis of the renal vein and liver metastasis; moreover, bulky mass caused compression of the other abdominal organs and displaced them from the physiological position. The pulmonary thromboembolism was excluded, although mild left basal pleural effusion with dysventilation of the left lower lobe was evident and related to bulky mass effect. The patient was on follow-up for breast cancer treated 3 years before by surgery followed by four cycles of epirubicin and cyclophosphamide-based adjuvant chemotherapy, paclitaxel for 12 weeks, radiotherapy, and tamoxifen-based hormonal therapy, with a residual ejection fraction of 35% probably as a consequence of anthracycline therapy. Previously, she underwent also total thyroidectomy for multinodular goiter. The selective arteriography, performed 24 h before surgery, showed a mass blood supply from the first three lumbar arteries and splenic artery short branches that were embolized by hemostatic agents. A cava filter was also placed. A total body bone scan was performed for staging and was negative for bone metastasis (Figure 2).

The patient underwent a laparotomic left radical nephrectomy with adrenalectomy and partial resection of psoas muscle and diaphragm, which were macroscopically involved by tumor, in order to achieve a radical surgery. Hilar and para-aortic lymphadenectomy were also carried out. Operative time was 200 min. Estimated blood loss was 550 cc. There were not any perioperative complications. No blood transfusion was needed. The patient was dismissed on post-operatory day 13.

Macroscopic examination showed a 200 × 150 × 90 mm grey-yellow, friable mass with capsular interruption and containing necrotic and hemorrhagic areas. Surgical margins were unclear at level of psoas and diaphragm resection. Histologically, the tumor was made up of nodules separated by sclero-hyaline septa and it was composed of small to medium-sized cells with oval nuclei, small nucleoli, granular chromatin, and scanty cytoplasm. The mitotic activity was high. On immunohistochemistry, the neoplastic cell resulted to be diffusely positive for CD99, focally positive for CD56, and focally and weakly positive for synaptophysin. The neoplastic cells were negative for cytokeratin (CKAE1/AE3), epithelial membrane antigen (EMA), S-100, leucocyte common antigen (LCA), CD2, CD20, CD79 alpha, CD117, LAT, CD10, MPO, TdT, CD68PGM1, NPM1, CD38, PAX5, CD31, CD34, and desmin. The proliferative index (KI67/MIB-1) was about 70%. The genetic analysis revealed the presence of EWSR1 rearrangement. The immuno-morphological analysis highlighted a malignant aggressive neoplasm with EWSR1 rearrangement, in keeping with EES. Lymph nodes were free of tumor. Four weeks after surgery, the patient underwent ESFT 2001 chemotherapy protocol, including 5 cycles of injectable vincristine (1.6 mg), ifosfamide (2.2 g), mesna (440 mg), and etoposide (110 mg). The protocol, the most suitable in this case, comprehensive of adriamycin in addition to vincristine et ifosfamide, was not administered due to the poor heart ejection fraction.

**Figure 3:**
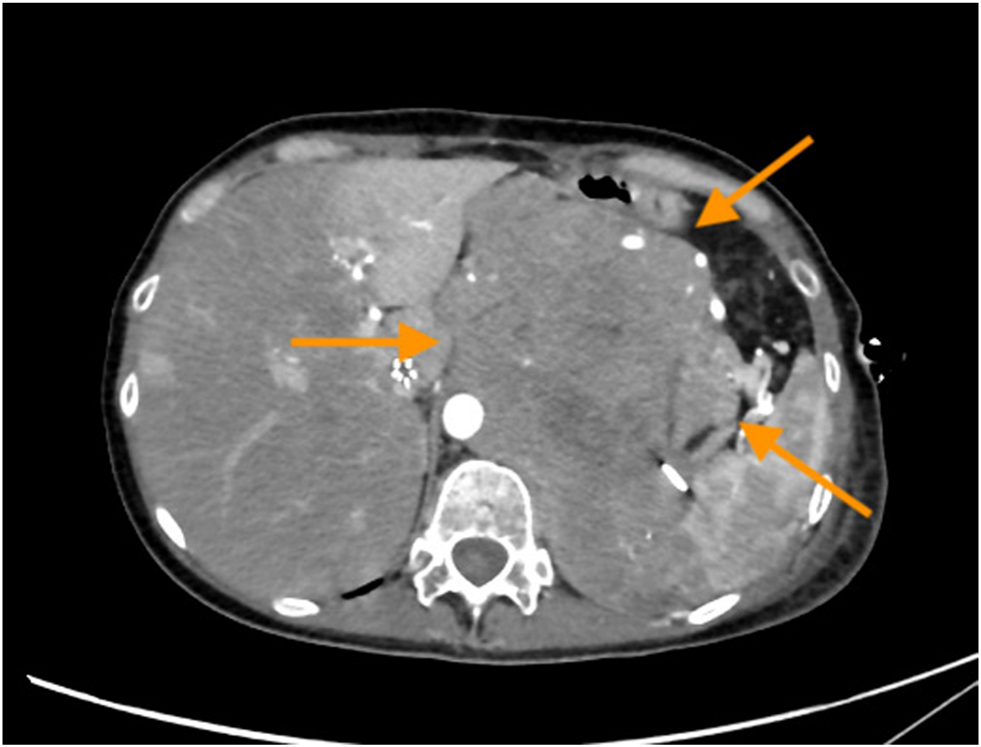
CT-scan, the imaging revealed a huge recurrence in the surgical site (arrows) with involvement of thoracic wall.

**Figure 4:**
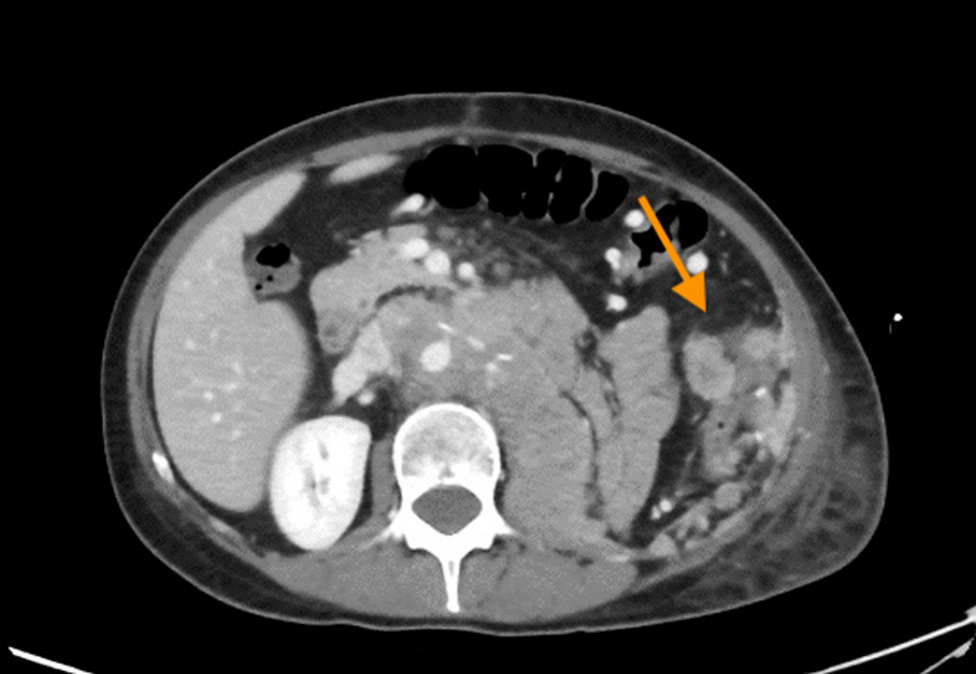
CT-scan, the imaging revealed a peritoneal carcinomatosis (arrow).


After the first cycle of chemotherapy, the patient required emergency care for dyspnea. An X-ray exam showed left pleural effusion and a chest drainage was placed. A CT scan disclosed a huge recurrence in the surgical site and a peritoneal carcinomatosis as shown in Figures 3 and 4. Patient refused a second cycle of chemotherapy and died four months after surgery.

**Informed consent:** Written informed consent was obtained from the patient for publication of relevant medical information and all of accompanying images within the manuscript.

## 3 Discussion

ES was first described by Stout in 1918 and later on in 1921 by James Ewing who characterized this tumor describing it in the diaphysis of long bones. ES in children and adolescents is defined as a bone tumor, which may occur at any site within the skeleton, but preferentially affects the trunk and the diaphysis of long bones [5]. However, in 15% of cases it may occur in extra-skeletal soft tissue. On the opposite, when diagnosed in adults, ES often affects soft tissues [6,7].

About 85–90% of EES cases present a somatic reciprocal *t* (11; 22)(q24; q12) chromosomal translocation, which fuses *EWSR1* to the *FLI1* ETS family gene to generate *EWSR1-FLI1* fusion transcripts which induce mitotic defects leading to genomic instability and subsequent malignant transformation [3,8,9].

A first challenge for this kind of lesion is the diagnosis. Clinical symptoms are not characteristic when present.

**Figure 5:**
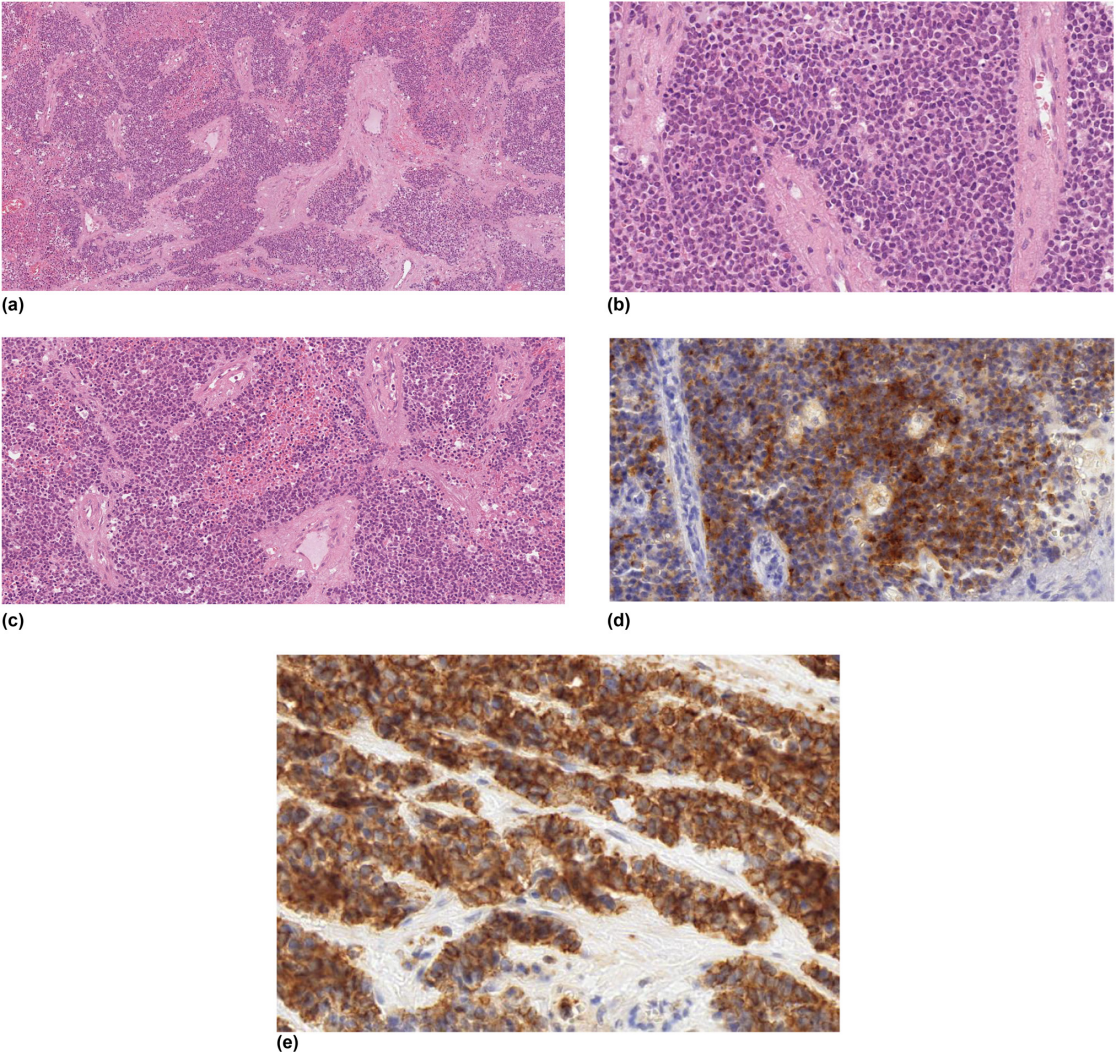
Histologic findings (a–e) revealed EES appearing as a poorly differentiated, small round blue cells tumor positive for the transmembrane glycoprotein CD99, vimentin, and CD 117 staining. Hematoxylin and eosin staining (a–c) show the lesion composed by small to medium-sized cells arranged in nodules separated by sclero-hyaline septa. Synaptophysin marker (d and e).

As in other pathologic conditions, radiological findings are usually not typical and present a wide spectrum of imaging features and metastatic patterns. The imaging features of primary EES on contrast-enhanced CT or magnetic resonance imaging (MRI) are bulky heterogeneous masses with frequent local invasion or with compression of adjacent organs. Retroperitoneal masses are usually large at diagnosis with 50% of lesions bigger than 20 cm [10]. Most cases show heterogeneous enhancement with large necrotic foci. Only rare cases present relatively well-defined margins, while local invasion to adjacent organs is commonly observed. The invasion of adjacent organs and nearby muscles is commonly noted in tumors of the abdomen, pelvis, and thorax as it was in our case [1,11,12,13,14]. The definitive diagnosis is histopathologic with immunohistochemical analysis. At the histological level, EES appears as poorly differentiated, small round blue cells tumor positive for the transmembrane glycoprotein CD99, vimentin, FLI1, CKAE1/AE3, and CD 117 staining and negative TLE and WT-1 [15,16,17,18] (Figure 5). The tumor cells have a pale-to-clear scanty cytoplasm and glycogen could be highlighted on PAS staining [17].

In recent literature, more and more cases of extra-skeletal sites in adults are reported. Probably, this increase is related to the availability of molecular diagnostic techniques and not to a true increase in its incidence [19].

ES of the kidney is a rare tumor in adults and it was described for the first time in 1975 by Seemayer and colleagues. Some authors suggested that the origin of ES in the kidney could be neural cells invaginated into the kidney during their development or embryonic neural crest cells migrating into the kidney and undergoing tumorigenesis [20]. The occurrence of EES in the kidney is uncommon and represents about 1% of all renal tumors, with less than 150 cases described in literature [21,22]. Male sex is more involved with a ratio M:F of 2:1.

At diagnosis, the most common symptom is pain (in 63%), followed by hematuria in 41% and a palpable mass in 25% [22]. In our case, the patient underwent chest-abdomen CT scan for persistent chest pain and suspicion of pulmonary thromboembolism.

Regarding renal ES (Table 1), 57% of patients had metastatic disease at the time of diagnosis [23]. Almost all of the patients (89%) who underwent biopsy had a metastatic disease at time of presentation, whereas about 1/3 of the patients (35%) who did not undergo biopsy had metastatic disease at the time of diagnosis.

**Table 1:** Renal Ewing Sarcoma/primitive neuroectodermal tumor (PNET)

Reference	Year	No of cases	Mean age (year)	Sex	Side	Symptoms at diagnosis	Mean size of tumor (cm)	Metastasis at diagnosis	Biopsy	Therapy	Median FU (months)	Outcome
Soni and Wei [38]	2017	1	28	F	Left	AbP, FP	14	IVC and left renal vein	No	RN + adjuvant chemotherapy	ND	ND
Teegavarapu et al. [39]	2017	13	33	11 M; 2 F	8 left; 5 right	6 FP, 2 AbP, 1 BP, 2 PM, 4 Hmt; 1 fv; 1 wt loss; 1 testicular swelling;	12	8 lung; 7 Rp; 1 liver; 1 LNs; 1 bone; 1 brain, 1 eyes, 1 adrenal, 1 pulmonary vasculature, 11 pts with metastasis	yes	9 RN + adjuvant chemotherapy; 4 chemotherapy	36.5	2 CFS; 1 OS
Abolhasani et al. [40]	2016	1	21	M	Right	FP, wt loss	18 × 8 × 5	No	No	RN	ND	ND
Abolhasani et al. [40]	2016	1	31	M	Right	Hmt	2 × 2 × 2	No	Yes	Neoadjuvant chemotherapy + RN	ND	ND
Abolhasani et al. [40]	2016	1	34	F	Left	Abd swelling, left lower chest pain, PM	ND	No	No	RN	ND	ND
Yamamoto et al. [41]	2015	1	35	M	Right	AbP, Hmt	15.5 × 13.5 × 10	IVC, Lung	Yes	RN + metastasectomy	27	OS
Chakrabarti et al. [42]	2015	1	24	M	Right	AbP, PM	6 × 5	No	No	RN + adjuvant chemotherapy	15	OS
Almeida et al. [43]	2014	1	19	M	Right	FP, fv, vomit	ND	Lung	Yes	chemotherapy	ND	ND
Liu et al. [44]	2014	1	37	M	Left	FP, Hmt	4 × 2.3 × 1.5	No	No	Open RNU + adjuvant chemotherapy	18	CFS
Hakky et al. [20]	2013	1	33	M	Left	nausea, vomit, FP	5.1 × 4.8 × 3.3	No	No	Robotic PN	12	CFS
Richey et al. [45]	2012	1	50	M	Right	FP, Hmt, PM	15.9 × 10.6	intrathoracic and Abd LNs, renal fat and vessel, lung, liver	Yes	chemotherapy	ND	ND
Tariq et al. [46]	2012	1	57	F	ND	ND	ND	Lung	ND	Multimodal	96	CFS
Alonso et al. [47]	2011	1	20	M	Left	Hmt	7	Lung, retroperitoneal carcinomatosis	No	Open RN + adjuvant chemotherapy	24	CFS
Alonso et al. [47]	2011	1	43	M	Right	Pneumonic process	ND	Lung, IVC	No	Open RN + thrombectomy + adjuvant chemotherapy	9	CFS
Pathak et al. [48]	2011	1	44	F	Right	PM	17.4 × 11.5 × 9.3	Renal vein, IVC, atrium	No	RN + thrombectomy	ND	ND
Mohsin et al. [49]	2011	1	26	M	Right	FP, AbP, Hmt, wt loss, PM	20 × 13 × 10	Lung, lymphadenopathy, renal vein	No	RN + adjuvant chemotherapy	10 days	OS
Mohsin et al. [49]	2011	1	25	M	Left	Hmt, FP, PM	15 × 11 × 8	Renal vein, lung, liver	No	RN	2	OS
Mohsin et al. [49]	2011	1	30	F	Right	Abd swelling, PM	18 × 12	Vertebral bodies	Yes	Chemotherapy	1	ND
Mohsin et al. [49]	2011	1	34	M	Right	Hmt, PM	17 × 10 × 9	Liver, lung	No	RN	14 days	OS
Wedde et al. [51]	2011	1	73	M	Right	Hydrocele	ND	No	Yes	RN + adjuvant chemotherapy	7	CFS
Badar et al. [50]	2010	1	13	F	Right	AbP, Hmt	6 × 9	Lung, liver	Yes	RN + adjuvant chemotherapy + RT	26	OS
Asiri and Al-Sayyad [52]	2010	1	11	M	Right	FP, Hmt, PM	10 × 9 × 11	No	No	RN + lymph node dissection + adjuvant chemotherapy	3	OS
Angel et al. [53]	2010	1	31	M	Left	FP, Hmt	7.9	No	No	RN	12	CFS
Ohgaki et al. [54]	2010	1	21	M	Right	AbP, hemorrhage	ND	No	No	RN	6	OS
Fergany et al. [55]	2009	1	31	M	Right	Hmt	16	Renal vein, IVC, lung	No	RN + lymph node dissection + IVC thrombectomy	24	CFS
Businger et al. [56]	2009	1	46	F	Right	AbP	5 × 12 × 16	Renal vein	No	Open retroperitonealectomy with RN + adjuvant chemotherapy	ND	ND
Ishii et al. [57]	2009	1	21	M	Right	ND	ND	No	No	RN	6	OS
Zhang et al. [58]	2009	1	41	M	ND	ND	ND	IVC	No	RN + adjuvant chemotherapy + RT	9	CFS
Ong et al. [59]	2008	1	21	F	Right	PM, pain, Hmt	12.5 × 9	Renal vein, IVC, atrium, lung	No	RN + thrombectomy	10	CFS
Koski et al. [60]	2008	1	78	F	Left	Syncope, respiratory failure, Hmt	9 × 9 × 8.7	Pulmonary embolus, renal vein, IVU, lymphadenopathy	No	RN + lymph node dissection + thrombectomy	2 weeks	OS
Chu et al. [61]	2008	1	14	F	Left	FP	ND	Renal vein, IVC, psoas, spinal canal, liver	Yes	Chemotherapy	22	OS
Chu et al. [61]	2008	1	16	F	Left	BP, decrease of sensation, PM	ND	Renal vein, IVC, spinal canal, L1, lung	Yes	Neoadjuvant chemotherapy + RN + thrombectomy + RT	4.5	CFS
Chu et al. [61]	2008	1	10	F	Right	Hmt	ND	Renal vein, IVC, atrium, lung	Yes	Neoadjuvant chemotherapy + RN + RT	24	CFS
Kang et al. [62]	2007	1	34	M	Left	Hmt, pain	8.6 × 6	Bone	Yes	RN	ND	ND
Moustafellos et al. [63]	2007	1	32	M	Right	AbP, FP	3.5 × 4.3 × 4	No	No	RN + adjuvant chemotherapy	36	CFS
Parada et al. [64]	2007	1	19	M	Left	FP, fv	7.5	ND	No	RN + adjuvant chemotherapy	ND	ND
Ellinger et al. [23]	2006	1	39	M	Left	Hmt, testicular pain, varicocele	12	Yes	No	RN + thrombectomy + splenectomy + adjuvant chemotherapy	6	CFS
Ellinger et al. [23]	2006	1	28	M	Right	FP, leg pain	9 × 5 × 5	Lung, renal vein, IVC, lymph nodes		RN + vena cava resection + adjuvant chemotherapy + RT	15	CFS
Saxena et al. [65]	2006	1	26	F	Left	Dispnea, nausea	18 × 14 × 13	Lung	Yes	RN + adjuvant chemotherapy	6	OS
Maeda et al. [66]	2006	1	6	F	Right	AbP, PM	5 × 4.5 × 4.5	No	No	RN + lymph node dissection + adjuvant chemotherapy	90	CFS
Erkiliç et al. [67]	2006	1	45	M	Left	FP, Hmt	ND	No	No	RN	12	CFS
Pomara et al. [68]	2004	1	27	F	Left	FP, Hmt	11 × 8 × 6	Renal vein	No	Surgery + adjuvant chemotherapy + RT	24	CFS
Sivaramakrishna et al. [69]	2003	1	27	M	Left	FP, PM	16 × 11	Renal vein	No	RN + adjuvant chemotherapy	9	CFS
Murphy et al. [70]	2003	1	26	M	Right	Bilateral FP	6	Renal vein, IVC	No	RN + thrombectomy + adjuvant chemotherapy	ND	ND
Wada et al. [71]	2003	1	23	F	Right	Fatigue, fv, FP, Hmt	13 × 10	Lung, renal vein IVC	No	RN + thrombectomy	12	CFS
Vicha et al. [72]	2002	1	9	F	Right	PM	15 × 14 × 11	No	No	RN + adjuvant chemotherapy	5	OS
Karnes et al. [73]	2000	1	28	M	Right	Hmt, BP	13 × 13	Renal vein + IVC	No	RN + adjuvant chemotherapy	12	CFS
Kuroda et al. [74]	2000	1	28	M	Left	AbP	7.4 × 5.7 × 7.6	No	No	RN	ND	ND
Herman et al. [75]	1997	1	17	ND	Right	Abd swelling, Hmt	ND	ND	No	RN	ND	ND
Fontaine et al. [76]	1997	1	42	M	ND	ND	ND	ND	ND	Surgery + RT + adjuvant chemotherapy	60	CFS
Mor et al. [77]	1994	1	61	ND	ND	ND	ND	ND	No	Surgery + adjuvant chemotherapy + RT	6	OS

FP, flank pain; AbP, abdominal pain; BP, back pain; PM, palpable mass; Hmt, hematuria; fv, fever; RN, radical nephrectomy; PN, partial nephrectomy; RNU, radical nephroureterectomy; RT, radiotherapy; IVC, inferior vena cava; LNs, lymph nodes; Rp, retroperitoneal; Abd, abdominal; wt, weight; CSF, cancer free survival; OS, overall survival.

In our case, the huge mass showed imaging features of malignant tumor, and for this reason, we performed a surgical treatment without biopsy. Due to bulky size and wide blood supply of the tumor, a preoperative renal angio-embolization was carried out in order to reduce the risk of intraoperative bleeding and to facilitate surgical procedure, thus decreasing operative time and perioperative morbidity. Other important benefits of preoperative embolization included the potential role of an early ligation of the renal vein before the renal artery has been fully controlled, according to the indications given by Robson. In literature, the real usefulness of preoperative renal embolization is still debated. However, a recent prospective, randomized study showed preoperative renal embolization to be a safe and well-tolerated procedure that allowed to reduce median blood loss in patients with huge kidney cancer who underwent embolization before nephrectomy compared to patients who did not undergo preoperative embolization [24].

Prognosis is poor in metastatic disease [25,26,27,28,29], whereas in cases of nonmetastatic lesions a survival benefit is reported from 18 to 51 months which is best related with negative surgical margins [30,31,32,33]. Also, age (≥40 years) and dimensions of tumor (larger diameter of at least 8 cm) are considered prognostic factors associated with poor cancer-specific survival rate [34]. Our case seemed to be one of them with poor prognosis related to its huge local extension in addition to metastasis.

Multimodal approach including surgery associated to the adjuvant chemotherapy is the standard therapeutic approach, with radiotherapy (RT) playing an optional role in localized and nonsurgical tumors. However, 32.5% of patients received only surgical treatment, while 13% received only chemotherapy. In the 87% of the patients who received surgical treatment, a nephrectomy was performed. About 5.5% of surgical patients received neoadjuvant chemotherapy, 47% adjuvant chemotherapy, and 15% adjuvant chemotherapy and radiotherapy. The mean overall survival (OS) in the group of patients treated by multimodal approach was 20.8 months, whereas that in the patients who received only surgery was 10.3 months [25,26,27,28,29,30,31,32].

The key point in the management of these tumors is to obtain a complete surgical debridement with clear negative margins, but this is affected by the disease stage at diagnosis [35,36]. In our case, the multimodal approach did not improve patient prognosis with a rapid onset of local recurrence and cancer-specific survival was 4 months. This may be due to unclear surgical margins at level of psoas and diaphragm. A reasonable therapeutic approach in case of doubt to obtain clear surgical margins could be the choice of neoadjuvant chemotherapy. An eventual response to it could be the permission to perform a surgical operation. However, early relapse, within 2 years of first diagnosis, is reported in 70% of cases. In 66% of relapsing disease, it occurs at distant sites in metastatic disease at diagnosis, while isolated local recurrence is described in 20% of cases and is more frequent in ES localized at diagnosis [37].

Table 2 shows the review of the literature concerning nonrenal extraosseous ES/PNET. In this type of tumor, 26% of patients had metastatic disease at the time of diagnosis. The ratio M:F is lower than that in renal ES and it is around 1.25:1. Thirty percent of the patients underwent surgery, 50% of whom received neoadjuvant chemotherapy, 25% adjuvant one. Radiotherapy was carried out in 56% of all patients. Differences between these two groups concern metastatic disease which is more common in the renal ES one at diagnosis. In this group, the percentage of patient treated by nephrectomy was significantly higher, while neoadjuvant chemotherapy associated with radiotherapy was used in a multimodal treatment in a low percentage of cases. On the other hand, patients with nonrenal extraosseous ES received often a multimodal treatment with neoadjuvant chemotherapy associated with radiotherapy. In this group, surgical approach is not the main treatment of choice.

**Table 2:** Nonrenal extraosseous ES/PNET

Reference	Year	No of cases	Mean age (year)	Sex	Site	Symptoms at diagnosis	Mean size of tumor (cm)	Metastasis at diagnosis	Biopsy	Therapy	Median FU (months)	Outcome
Singla et al. [78]	2016	1	26	M	Lumbar epidural space	BP	ND	No	No	Laminectomy + adjuvant chemotherapy + RT	12	CFS
Lu et al. [79]	2015	1	40	F	Rp in the right hepatorenal recess	FP	10	ND	No	Tumor resection	12	OS
Mohsin et al. [49]	2011	1	29	M	Prostate	Burning micturation, PM	ND	Bladder, lymphadenopathy, lung, pleural	Yes	Chemotherapy	4	ND
Mohsin et al. [49]	2011	1	20	F	Right adrenal	FP, anorexia, wt loss, PM	ND	Lung, ascite	Yes	Chemotherapy	ND	ND
Yip et al. [80]	2009	1	28	F	Near the vaginal introitus	ND	2	ND	No	RT	12	OS
García-Morena Nisa et al. [81]	2007	1	21	F	Rp and retrodiaphragmatic	FP, AbP, PM	21 × 17 × 12	No	Yes	Neoadjuvant chemotherapy + mass excision + adjuvant chemotherapy	16	CFS
Venkitaraman et al. [82]	2007	19	21	12 M; 7 F	Thorax (4), upper extremity (3), Rp (2), paraspinal (3), Pelvis (3), lower extremity (4)	ND	10.5	4		Local 15 chemotherapy + 3 surgery 9 RT Metastatic RT + chemotherapy	12	7 CFS; 4 OS
Ellinger et al. [23]	2006	1	72	M	Bladder	Hmt, oliguria	ND	Prostate, Abd wall, peritoneum	No	TURB then surgery	2	OS
Thebert et al. [83]	1993	1	22	F	Rp	BP, AbP, PM	18 × 15 × 22	ND	No	Tumor resection + RN + LNs dissection	ND	ND

FP, flank pain; AbP, abdominal pain; BP, back pain; PM, palpable mass; Hmt, hematuria; LNs, lymph nodes; Rp, retroperitoneal; wt, weight; RT, radiotherapy; CSF, cancer free survival; OS, overall survival.

## 4 Conclusion

Reporting this case, we would point out that in presence of a renal mass, especially if huge, it has to be borne in mind that EES can occur primarily in the kidney. The local invasion should be well-evaluated before the surgery because, in case of EES, only a complete surgical ablation of the tumor could improve cancer-specific survival.

In adult, advanced ES is a dramatic condition with inauspicious outcome. Often, successful rate of surgical treatment may be affected by complexity to obtain negative surgical margins due to disease extension. Due to this, surgery in advanced disease may be considered as an important step of multimodal treatment.
